# The treatment of post-hysterectomy vaginal vault prolapse: a systematic review and meta-analysis

**DOI:** 10.1007/s00192-017-3493-2

**Published:** 2017-10-16

**Authors:** Anne-Lotte W. M. Coolen, Bich Ngoc Bui, Viviane Dietz, Rui Wang, Aafke P. A. van Montfoort, Ben Willem J. Mol, Jan-Paul W. R. Roovers, Marlies Y. Bongers

**Affiliations:** 1 Department of Obstetrics and Gynecology, Máxima Medical Centre, De Run 4600, 5500 MB Veldhoven, The Netherlands; 2 Department of Obstetrics and Gynecology, Catharina Hospital, Michelangelolaan 2, 5623 EJ Eindhoven, The Netherlands; 3 Robinson Research Institute, Adelaide Medical School, University of Adelaide, Adelaide, SA Australia; 4 Department of Obstetrics and Gynaecology, Maastricht University, Grow School for Oncology and Developmental Biology, Minderbroedersberg 4, 6211 LK Maastricht, The Netherlands; 5 Department of Gynecology and Obstetrics, Academic Medical Centre Amsterdam, Meibergdreef 9, 1105 AZ Amsterdam, The Netherlands

**Keywords:** Vaginal vault prolapse, Pelvic organ prolapse, Treatment, Surgical treatment, Sacrocolpopexy, Trans vaginal mesh, Sacrospinous fixation

## Abstract

**Introduction and hypothesis:**

The treatment of post-hysterectomy vaginal vault prolapse (VVP) has been investigated in several randomized clinical trials (RCTs), but a systematic review of the topic is still lacking. The aim of this study is to compare the effectiveness of treatments for VVP.

**Methods:**

We performed a systematic review and meta-analysis of the literature on the treatment of VVP found in PubMed and Embase. Reference lists of identified relevant articles were checked for additional articles. A network plot was constructed to illustrate the geometry of the network of the treatments included. Only RCTs reporting on the treatment of VVP were eligible, conditional on a minimum of 30 participants with VVP and a follow-up of at least 6 months.

**Results:**

Nine RCTs reporting 846 women (ranging from 95 to 168 women) met the inclusion criteria. All surgical techniques were associated with good subjective results, and without differences between the compared technique, with the exception of the comparison of vaginal mesh (VM) vs laparoscopic sacrocolpopexy (LSC). LSC is associated with a higher satisfaction rate. The anatomical results of the sacrocolpopexy (laparoscopic, robotic [RSC]. and abdominal [ASC]) are the best (62–91%), followed by the VM. However, the ranges of the anatomical outcome of VM were wide (43–97%). The poorest results are described for the sacrospinal fixation (SSF; 35–81%), which also correlates with the higher reoperation rate for pelvic organ prolapse (POP; 5–9%). The highest percentage of complications were reported after ASC (2–19%), VM (6–29%), and RSC (54%). Mesh exposure was seen most often after VM (8–21%). The rate of reoperations carried out because of complications, recurrence prolapse, and incontinence of VM was 13–22%. Overall, sacrocolpopexy reported the best results at follow-up, with an outlier of one trial reporting the highest reoperation rate for POP (11%). The results of the RSC are too small to make any conclusion, but LSC seems to be preferable to ASC.

**Conclusions:**

A comparison of techniques was difficult because of heterogeneity; therefore, a network meta-analysis was not possible. All techniques have proved to be effective. The reported differences between the techniques were negligible. Therefore, a standard treatment for VVP could not be given according to this review.

**Electronic supplementary material:**

The online version of this article (10.1007/s00192-017-3493-2) contains supplementary material, which is available to authorized users

## Introduction

More than 40% of women aged 40 and older have pelvic organ prolapse (POP) [1]. The incidence of vault prolapse requiring surgery has been estimated to be 36 per 10,000 women years [2]. The risk of prolapse following hysterectomy is 5.5 times higher in women whose initial indication for hysterectomy was pelvic organ prolapse as opposed to other indications [3]. The number of women with a symptomatic POP who seek medical help is increasing [4]. Vaginal vault prolapse (VVP) is often associated with other compartment defects (cystocele, rectocele, or enterocele), which makes it a challenging condition to treat [5]. There is a growing recognition that adequate support for the vaginal apex is an essential component of a durable surgical repair for women with advanced prolapse [3]. Because of the significant contribution of the apex to vaginal support, anterior and posterior vaginal repairs may fail unless the apex is adequately supported [6].

Current treatment options for VVP include pelvic floor muscle training, use of pessaries, and surgery [7]. More than 20 different surgical procedures for correcting VVP have been reported [6, 8, 9]; abdominal sacrocolpopexy by laparotomy (ASC), laparoscopy (LSC) and robotics (RSC), using xenograft, polypropylene, abdominal fascia or fascia lata. Sacrospinal fixation (SSF) and transvaginal mesh (VM) are the most frequently used surgical techniques. The best treatment for post-hysterectomy VVP remains controversial. Maher [6] reviewed the management of apical prolapse, but management of uterine descent and VVP were not separately investigated.

The treatment of post-hysterectomy VVP has been investigated in several randomized clinical trials, but a systematic overview of the topic is still lacking. We compared the effectiveness of post-hysterectomy vaginal vault treatments in a systematic review and meta-analysis, combined with a network plot, thus utilizing the most reliable evidence coming from randomized controlled trials.

## Materials and methods

### Types of studies

We searched the literature for randomized controlled trials (RCTs) in which any treatment was compared with any other treatment for VVP. Treatment was defined as any treatment to treat a post-hysterectomy vaginal vault prolapse. Trials reporting on the objective and/or subjective outcome of VVP treatments were eligible if they reported on at least 30 participants and a follow-up of at least 6 months. Quasi-randomized studies and cross-over studies were not included. The effectiveness of the treatments was evaluated through the objective (anatomical) results and/or the subjective (quality of life and satisfaction) results.

A systematic review of the literature on the treatment of post-hysterectomy VVP was performed according to the Preferred Reporting Items for Systematic Reviews and Meta-Analyses (PRISMA) checklist [10]. Studies were identified by searching PubMed (MEDLINE) and Embase, using the search term “vaginal vault prolapse.” The last literature search was run on 25 April 2017. An overview of our full electronic search strategy is presented in Appendix 1. Narrowing down the search by adding the search terms “therapy” or “systematic review,” resulted in the loss of relevant articles. We therefore chose a broad search with the term “vaginal vault prolapse.” Reference lists of relevant articles identified were checked for additional articles. No restrictions on language or publication year were applied, and foreign-language papers were translated. We did not impose any other limits on any of the searches.

### Types of participants

Eligible trials included women seeking treatment for a symptomatic primary VVP, defined as a post-hysterectomy prolapse of the apical compartment. If trials reported on a combination of uterine prolapse and (non-)post-hysterectomy VVP, they were excluded when no subgroup analysis was performed on the group with a VVP.

### Types of interventions

Eligible trials compared different types of treatment for VVP, including physiotherapy, pessary treatment, abdominal surgery (open, laparoscopic or robotic), vaginal surgery, native tissue repair, and mesh surgery.

### Types of outcomes

The primary outcomes of the review are the objective (anatomical) and subjective ([disease specific] quality of life) outcome of VVP treatments. The objective outcome was defined as the assessment of POP by a validated staging system, i.e., Pelvic Organ Prolapse Quantification System (POP-Q) [11] or the Baden–Walker system [12]. The subjective outcome was defined as the assessment of subjective symptoms resulting from POP by validated questionnaires.

Other outcomes were follow-up time, blood loss during surgery, operating time, length of hospital stay, complications, any recurrent prolapse according to the POP-Q classification, repeat surgery for prolapse, mesh erosion and exposure, dyspareunia, and de novo incontinence. We also collected data about any other reported anatomical outcome, success rates and its definitions, and items of the composite score of Barber (recurrent pelvic organ prolapse beyond the hymen in the apical compartment, with bothersome bulge symptoms, and re-interventions). However, data of the Barber’s criteria were not available in many publications; therefore, we could not report these data in this review. We looked for outcomes that could be pooled for meta-analysis, and if pooling was not possible, data were reported in a table to create a clear overview of all the different outcome measurements of the trials.

Complications and mortality were recorded to assess the safety of the procedures. We classified the complications according to the Clavien–Dindo complication classification, to compare the complications of the included trials. This classification consists of four severity grades of complications [13]. Complications were categorized into grade 1 to 5 (grade 1: requires no treatment; grade 2: requires drug therapy; grade 3: requires a procedure or intervention; grade 4: IC/ICU organ or system dysfunction; grade 5: death), and complications of grade 3–5 were documented.

### Data collection

**Fig. 1:**
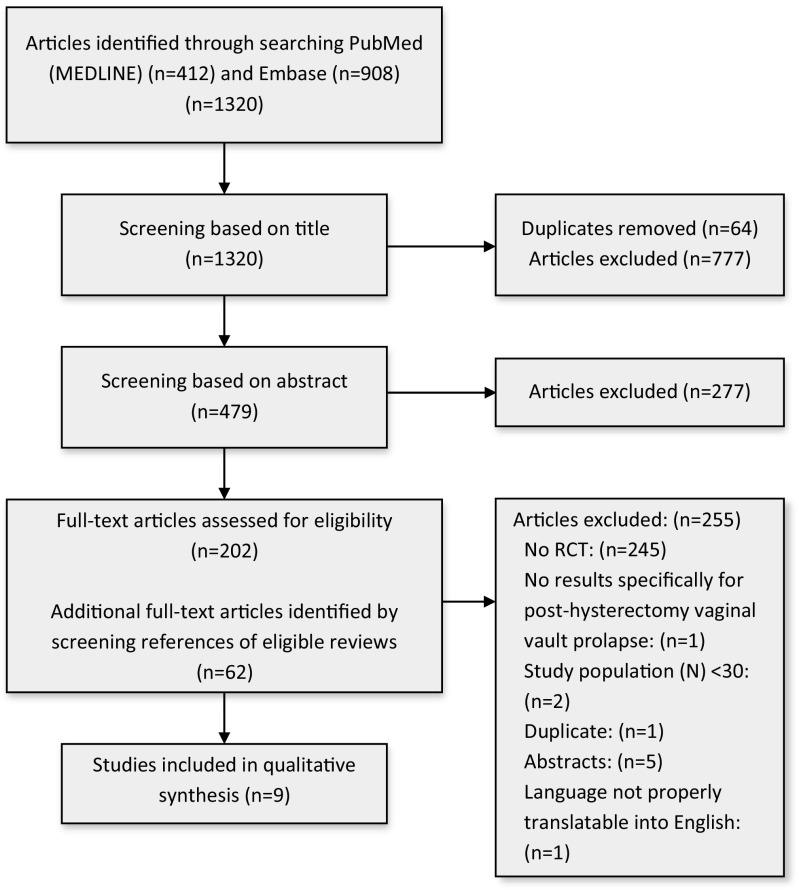
Preferred Reporting Items for Systematic Reviews and Meta-Analyses (PRISMA) flow chart

Titles and abstracts were assessed for eligibility by two independent reviewers (ALC and BNB). Disagreements were referred to a third reviewer (MYB or VD) to reach consensus. Data extraction was independently conducted by two authors (ALC and BNB) and recorded in a predefined data extraction sheet. The selection process can be referred to in the PRISMA flow chart (Fig. 1).

Two reviewers (ALC and BNB) independently assessed the quality of the trials included utilizing the Cochrane Collaboration’s tool for assessing risk of bias described in the Cochrane Collaboration Handbook [14]. Disagreements were discussed with a third reviewer (MYB) to reach consensus (Appendix 1).

### Data extraction and management

Data were extracted on type of intervention(s), number and age of trial participants, the trial’s inclusion and exclusion criteria, the follow-up duration, type of treatment, and type of outcome measure. Our outcome measure is the comparison of the objective and subjective outcomes of the trial interventions. Other extracted parameters are the language of the article, blinding, baseline characteristics, details of the intervention, complications, adverse events, repeat surgery, recurrent prolapse, and loss to follow-up.

### Assessment of risk of bias in the studies included

**Fig. 2:**
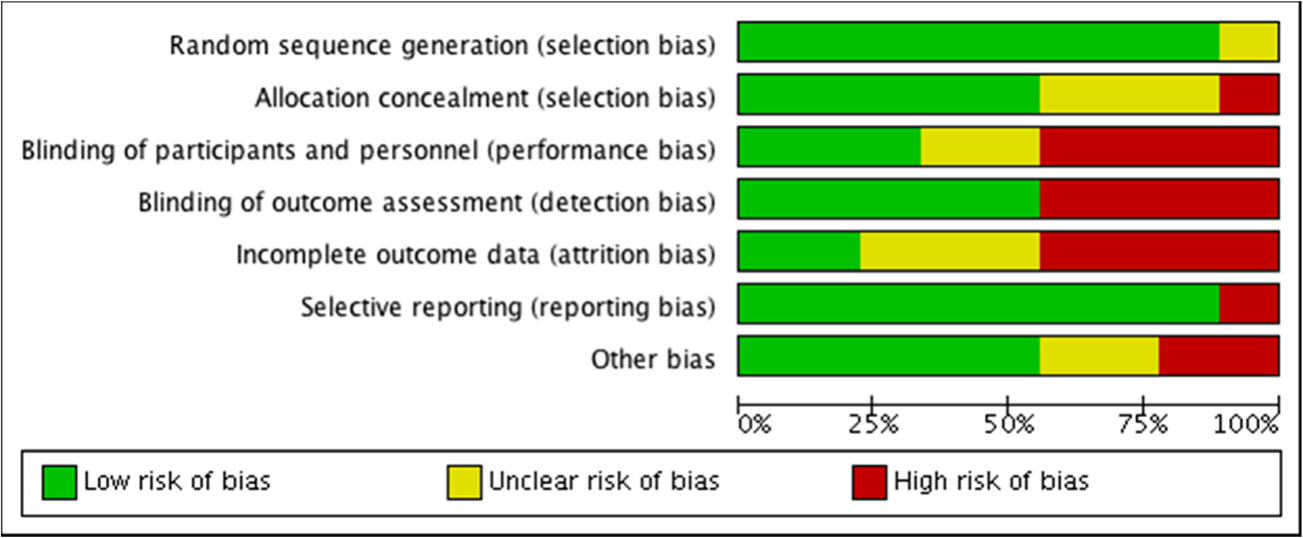
Risk of bias graph: review authors’ judgements about each risk of bias item presented as percentages across all the studies included

**Fig. 3:**
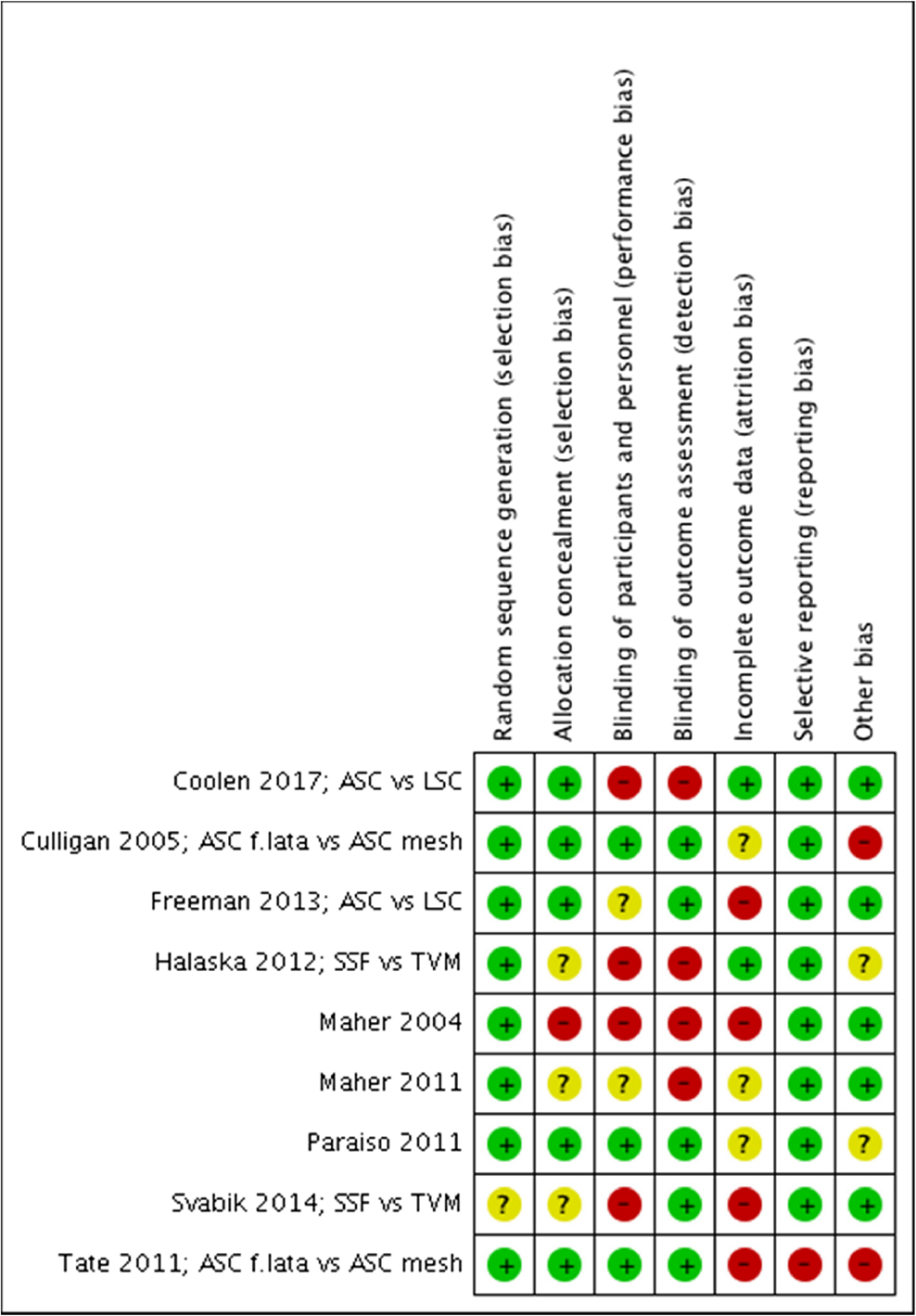
Risk of bias summary: review authors’ judgements about each risk of bias item for each study included

Risk of bias was assessed by using the Cochrane “risk of bias” assessment tool [14] to assess selection (random sequence generation and allocation concealment); performance (blinding of participants and personnel); detection (blinding of outcome assessors); attrition (incomplete outcome data); reporting (selective reporting); and other bias. We presented the conclusions in the “risk of bias” tables (Figs. 2, 3).

### Analysis

We created a network plot to illustrate the geometry of the network of the treatments included by using “mvmeta” package in Stata software (version 12.0; Stata Corp, College Station, TX, USA) [15]. For dichotomous data, we used the numbers of events in the control and intervention groups of each study to calculate Mantel–Haenszel odds ratios (ORs). For continuous data, if all studies reported exactly the same outcomes, we calculated the mean difference (MDs) between treatment groups. We presented 95% confidence intervals for all outcomes. We analyzed the data on an intention-to-treat basis (once randomized to an intervention, the participants are analyzed in that intervention and analysis includes all randomized participants) as far as possible. Review manager 5.3 was used for meta-analyses.

We considered whether the clinical and methodological characteristics of the studies included were sufficiently similar for meta-analysis to provide a clinically meaningful summary. We assessed statistical heterogeneity by measuring the I^2^. An I^2^ measurement greater than 50% was taken to indicate substantial heterogeneity [14], and a random-effects calculation was undertaken to express greater uncertainly by widening the confidence intervals.

## Results

### Study selection

The search of PubMed and Embase resulted in 1,320 citations (Fig. 1), 1,256 of which remained after undoubling. After screening of titles and abstracts, 1,054 articles were excluded, whereas 202 full text articles were assessed for eligibility. Screening on the title and abstract of references of eligible articles resulted in 62 additional eligible full-text articles. Out of 264 full-text articles, 255 were excluded after reading full text articles, whereas 9 RCTs were included in the systematic review.

### Characteristics of the studies included

All studies were randomized controlled trials level 1B according to the Oxford (UK) CEBM levels of evidence, and written in English, with a follow-up ranging from 12 to 60 months.

### Participants

The RCTs included involved 846 participants operated on for VVP. The main inclusion criteria entailed symptomatic vault prolapse indicated for surgical repair. The mean age of the participants in the RCTs ranged from 57 to 66 years. All participants had (post-hysterectomy) VVP with or without concomitant cystocele and/or rectocele. The mean preoperative stage of pelvic organ prolapse ranged from stage 2 (7 RCTs, *n* = 668) to 3 (2 RCTs, *n* = 178). The mean parity ranged from 2 to 3. Furthermore, the mean body mass index ranged from 25.3 to 29 kg/m^2^.

### Interventions

**Fig. 4:**
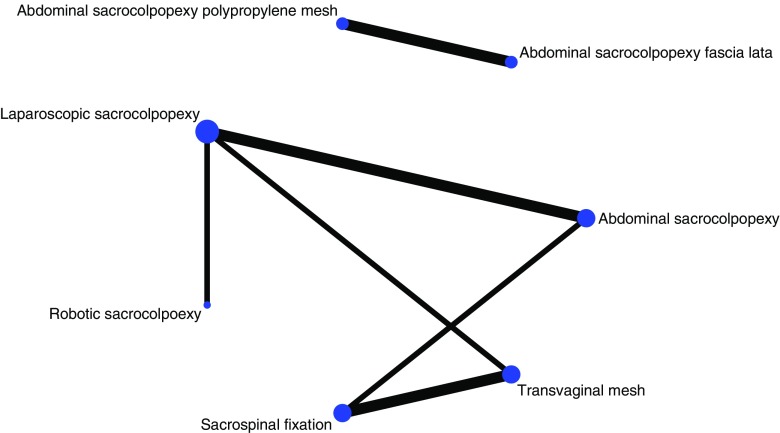
Network plot

A network plot was constructed (Fig. 4) to illustrate the geometry of the network of the treatments included. Two studies reported on ASC vs LSC [16, 17]. Two papers also reported on ASC and randomization between polypropylene and cadaveric fascia lata [18, 19]. Another trial compared ASC with SSF [20]. Two RCTs were randomized between SSF and VM (Total Prolift, Gynecare, Ethicon) [21, 22]. VM (Total Prolift, Gynecare, Ethicon) was compared with LSC in another trial [23], and LSC was compared with RSC [24] in one of the papers. The study by Tate et al. [18] was a report of the 5-year follow-up results of the same trial as Culligan et al. [19], who reported 1-year follow-up results.

### Follow-up time

Six of the studies included had a follow-up time of 1 year [16, 17, 19, 21–23]. In 2 trials, the follow-up time was 2 years [20, 23] and in 1 trial [18] a follow-up time of 5 years was administered.

### Outcomes

All 9 studies reported data in a form suitable for analysis on at least objective or subjective outcomes.

All trials reported objective outcomes: 1 study used the Baden–Walker staging system [20] and 8 studies used the POP-Q classification [16–19, 21–24]. Criteria for success were “no prolapse POP-Q ≥ stage 2” in 3 studies [19, 21, 23], or no prolapse Baden–Walker > grade 1 [20]. Composite scores according to Barber et al. [25] were used by Coolen et al. [16], Tate et al. [18], and Svabik et al. [22], although the items of the combinations were different. In 2 papers success was not defined [17, 24], but in both trials the POP-Q results were reported (Table 1).

**Table 1:** Objective anatomical outcome of randomized trials comparing treatments for vaginal vault prolapse

Reference	Number of patients (umber lost to follow-up if known)	Follow-up (months)	Intervention	Assessment of subjective outcome	Criteria for success	Objective success rate (%)	Re-operation (%)
[20]	95 (6)	ASC 24 (6–60) and SSF 22 (6–58)	ASC vs SSF	Baden–Walker	No prolapse grade 2	85 vs 81, *p* = 0.78	
[19]	100 (11)	12	ASC: fascia lata vs polypropylene mesh	POP-Q	No prolapse ≥stage 2	68 vs 91, *p* = 0.007	
[18]	100 (31)	60	ASC: fascia lata vs polypropylene mesh	POP-Q	Objective success: no prolapse ≥stage 2 Clinical success: no bulge or prolapse symptoms and point C <^1^/_2_TVL or any POP-Q point ≤0	62 vs 93, *p* = 0.02 90 vs 97, *p* = 0.61	
[17]	53	12	ASC vs LSC	POP-Q	Not defined	No difference	0 vs 1
[16]	74	12	ASC vs LSC	POP-Q	No prolapse beyond the hymen, no bulge symptoms, and no repeat surgery	84 vs 89	1 vs 5 (RR 4, 95% CI 0.84–5.73)
[23]	108 (3)	24	LSC vs VM	POP-Q	Stage 0 or 1 prolapse at all vaginal sites	77 vs 43, *p* < 0.001	
[24]	78 (17)	12	LSC vs RSC	POP-Q	Not defined	91 vs 88, NS (stage 0–1) 9 vs 12 NS (stage 2)	
[21]	168 (17)	12	SSF vs VM	POP-Q	No prolapse ≥stage 2	61 vs 83	
[22]	70 (0)	12	SSF vs VM	POP-Q	Point Ba, C or Bp <0 and translabial ultrasound: bladder descent <10 mm below the lower margin of the symphysis pubis on maximum Valsalva	35 vs 97, *p* < 0.001	

*ASC* abdominal sacrocolpopexy, *LSC* laparoscopic sacrocolpopexy, *SSF* sacrospinous fixation, *RSC* robotic sacrocolpopexy, *VM* total vaginal mesh

Seven reported the subjective success by using validated questionnaires (Table 2) [16, 17, 20–24]. Many different questionnaires were used to assess the subjective outcome (Table 2). Tate et al. [18] and Culligan et al. [19] did not report the subjective data they collected (according to their methods); however, as part of a combined outcome measurement, Tate et al. did report some subjective data of their population.

**Table 2:** Subjective outcome of randomized trials comparing treatments for vaginal vault prolapse

Reference	Number of patients (number lost to follow-up if known)	Follow-up (months)	Intervention	Assessment of subjective outcome	Outcome(s)	Result(s) (%)
[20]	95 (6)	Mean ASC 24 (6–60) and SSF 22 (6–58), *p* = 0.65	ASC vs SSF	UDI-6, IIQ, SF-36, modified sexual function questionnaires, VAS for patient satisfaction	Subjective success rate Patient satisfaction	94 vs 91, *p* = 0.19 85 vs 81, *p* = 0.78
[19]	100 (11)	12	ASC: fascia lata vs polypropylene mesh	No outcome	No outcome	No outcome
[18]	100 (31)	60	ASC: fascia lata vs polypropylene mesh	Clinical success: no bulge or prolapse symptoms and point C <^1^/_2_TVL or any POP-Q point ≤0	Combined subjective and objective outcome	90 vs 97, *p* = 0.61
[17]	53 (6)	12	ASC vs LSC	PGI-I, P-QOL, SF-36	Subjective outcome Quality of life	PGI-I score 1 and 2 combined 90 vs 80 No difference
[16]	74 (1)	12	ASC vs LSC	UDI, DDI, IIQ, PGI-I	Subjective outcome	No difference
[23]	108 (3)	24	LSC vs VM	APFQ, P-QOL, VAS for patient satisfaction	Symptomatic prolapse Mean patient satisfaction Subjective outcome	2 vs 7, *p* = 0.18 OR 4.75 (95% CI 2.06–10.98) 87 ± 21 vs 79 ± 20, *p* = 0.002 Mean difference 8.09 (95% CI, 0.20 –15.98) No difference
[24]	78 (17)	12	LSC vs RSC	PFDI-20, PFIQ-7, PISQ, EQ-5D	Subjective outcome Quality of life	No difference No difference
[21]	168 (17)	12	SSF vs VM	PISQ, UIQ, CRAIQ, POPIQ	Subjective outcome Quality of life	No difference No difference
[22]	70 (0)	12	SSF vs VM	ICIQ-SF, PISQ-12, POPDI, UDI, CRADI	Subjective outcome	No difference

*VM* total vaginal mesh, *PGI-I* patient global impression of improvement, *P-QOL* Perceived Quality of Life Scale, *SF-36* Short Form Health Survey, *UDI* Urogenital Distress Inventory, *DDI* Defecatory Distress Inventory, *IIQ* Incontinence Impact Questionnaire, *PISQ* Pelvic Organ Prolapse/Urinary Incontinence Sexual Questionnaire, *EQ-5D* Euroqol Questionnaire, *APFQ* Australian Pelvic Floor Questionnaire, *P-QOL* prolapse quality of life questionnaire, *VAS* Visual Analog Scale, *POPIQ* Pelvic Organ Prolapse Impact Questionnaire, *ICIQ-SF* International Consultation on Incontinence Questionnaire - Short Form, *POPDI* pelvic organ prolapse distress inventory, *CRADI* Colo-Rectal-Anal Distress Inventory, *UIQ* urinary impact questionnaire, *CRAIQ* Colorectoanal Impact Questionnaire

The publication of Tate et al. [18] reports on the 5-year follow-up of the same population as in the paper by Culligan et al. [19]. However, we decided to include both papers, as the main focus of each paper is a different outcome. They both report on anatomical outcome (no prolapse stage 2 or more). As this outcome is one of the primary outcomes of our review, and the difference in follow-up time is illustrative for this outcome, we decided to include this outcome for both papers. The other outcome on which they both report is complications, although different complications are reported; in the paper by Culligan et al. [19] all complications up to 1 year are reported and the paper by Tate et al. [18] only reports on mesh exposure.

### Risk of bias

The assessment of risk of bias in the studies included is presented in Figs. 2 and 3.

### Allocation

All trials were randomized trials and used adequate methods of allocation concealment [16–24], for example, randomization by sealed envelopes or computer-generated randomization. In 4 studies, block randomization was used [17–19, 24]. Inclusion of these 9 trials with well-performed randomization, resulted in a low risk of selection bias. However, in 1 of these trials [19], 4 participants received the other intervention (polypropylene mesh instead of fascia lata) and were not analyzed using the intention-to treat principle, as they were analyzed in the mesh group.

### Performance and detection bias

In some trials, blinding is very difficult because the type of incision is very different. However, patients were blinded in 3 trials [18, 19, 24] and in 1 trial patients were blinded during their admission [17]. The operating staff could not be blinded, although the ward staff were blinded in 2 trials [17, 18].

Blinding of outcome assessment at the follow-up consult was performed in 5 studies [17–19, 22, 24]. In 1 study the observer was an independent researcher, who was not blinded [16]. Three studies did not report any blinding of the outcome observation [20, 21, 23].

### Incomplete outcome data

In 7 studies [16–19, 21, 22, 24], follow-up rates were described. The follow-up rates varied within the range 69–97% with different follow-up periods. However, only 2 trials specified the reasons for loss to follow-up and looked at patient characteristics of responders and nonresponders [16, 18], which were balanced between groups. It is unclear if missing data were imputed in any of the studies, which results in a risk of bias.

### Reporting bias

Primary and secondary pre-specified outcomes were reported in 9 papers [16–24]. However, the data of several outcomes were not available to be used in a meta-analysis. Culligan et al. [19] and Tate et al. [18] did not report on the quality-of-life data they collected. Reasons for not reporting these data are not described.

### Confounders

Baseline characteristics able to act as confounders were reported in 9 studies [16–24]. However, Tate et al. [18] described only the preoperative POP-Q scores of the population. Significance between the two groups is not relevant as all trials were randomized properly (Appendix 3). Therefore, the risk of confounders is low.

### Other risk of bias

Other sources of bias were not found in any of the studies. However, funding was not described in all trials [18, 22]. Two trials were funded [17, 24], although these funding sources were not industry-driven.

### Anatomical outcome

Objective success rates according to the POP-Q or Baden–Walker classification, could be extracted from 8 studies (*n* = 793). All trials used their own definition of anatomical success. Success rates ranged from 62 to 93% for ASC (*n* = 284), 77 to 91% for LSC (*n* = 128), 35 to 81% for SSF (*n* = 165), 43 to 97% for VM (*n* = 176), and was reported to be 88% for RSC (*n* = 40; Table 1).

### Subjective outcome on urogenital symptoms and quality of life

Subjective outcomes could be extracted from 9 studies (*n* = 846). Many different questionnaires were used to assess subjective outcome. No significant differences were seen for subjective success and quality of life. Only 1 trial (*n* = 108) showed a higher satisfaction score in the LSC group compared with the VM group (Table 2).

### Complications

The most reported complications were classified as grade 2 and grade 3 complications (Table 3 and Supplementary Table 5).

**Table 3:** Clinical outcome

Reference	[20]	[19]	[18]	[17]	[16]	[23]	[24]	[21]	[22]
Comparison	ASC vs SSF	ASC: fascia lata vs polypropylene mesh	ASC: fascia lata vs polypropylene mesh	ASC vs LSC	ASC vs LSC	LSC vs VM	LSC vs RSC	SSF vs VM	SSF vs VM
Operative time (min)									
Mean (range)	106 ± 37 (45–100) vs 76 ± 42 (26–300) (*p* < 0.01)	233.4 ± 66.9 vs 227.3 ± 63.3 (*p* = 0.40)	Not mentioned	131 ± 44 vs 144 ± 28 (*p* = 0.24)	113 (68–180) vs 125 (85–240) (*p* = 0.31)	97 (36–280) vs 50 (30–96) (*p* < 0.001)	199 ± 46 (109–329) vs 265 ± 50 (191–381) (*p* < 0.001)	80 (15–50) vs 65 (35–166) (*p* = 0.001)	Not mentioned
Estimated blood loss (ml)									
Mean (range)	362 ± 239 (100–1,100) vs 306 ± 201 (100–1,000) (*p* = 0.22)	264.7 ± 261.4 vs 247.2 ± 148.4 (*p* = 0.68)	Not mentioned	240.4 ± 231.7 vs 56.15 ± 34.3 (*p* < 0.01)	205 (10–650) vs 86 (0–1,200) (*p* < 0.01)	100 (20–300) vs 150 (21–500) (*p* = 0.004)	Not mentioned	110 (10–528) vs 120 (10–814) (*p* = 0.39)	Not mentioned
Hospital stay (days)									
Mean (range)	5.4 ± 2.2 (3–16) vs 4.8 ± 1.4 (3–10) (*p* = 0.16)	Not mentioned	Not mentioned	4.1 ± 1.6 vs 3.2 ± 1.1 (*p* = 0.02)	4.3 (2–12) vs 2.4 (1–4) (*p* < 0.01)	2 (2–10) vs 3 (2–6) (*p* = 0.01)	1.4 ± 0.5 (0.6–2.7) vs 1.8 ± 1.5 (0.8–10) (*p* = 0.17)	Not mentioned	Not mentioned
Complications, % (n/m)^a^									
Grade 1	0 vs 2.1 (1/48)	0 vs 0	0 vs 0	0 vs 0	0 vs 0	0 vs 0	0 vs 2.9 (1/35)	6.8 (5/73) vs 0	0 vs 2.8 (1/36)
Grade 2	2.1 (1/47) vs 0	4.3 (2/46) vs 5.6 (3/54)	0 vs 0	0 vs 0	2.7 (1/37) vs 5.6 (2/36)	3.8 (2/53) vs 10.9 (6/55)	9.1 (3/33) vs 28.6 (10/35)	6.9 (5/73) vs 8.9 (7/79)	0 vs 0
Grade 3	10.6 (5/47) vs 6.3 (3/48)	10.9 (5/46) vs 25.9 (14/54)	2.3 (1/44) vs 4.4 (2/45)	7.4 (2/27) vs 7.7 (2/26)	13.5 (5/37) vs 5.6 (2/36)	9.4 (5/53) vs 18.2 (10/55)	9.1 (3/33) vs 22.9 (8/35)	9.6 (7/73) vs 34.2 (25/73)	0 vs 5.6 (2/36)
Grade 4	0 vs 0	0 vs 0	0 vs 0	0 vs 0	0 vs 0	0 vs 0	0 vs 0	0 vs 0	0 vs 0
Grade 5	0 vs 0	0 vs 0	0 vs 0	0 vs 0	2.7 (1/37) vs 0	0 vs 0	0 vs 0	0 vs 0	0 vs 0

^a^Grade 1: requires no treatment; grade 2: requires drug therapy; grade 3: requires a procedure or intervention; grade 4: IC/ICU organ or system dysfunction; grade 5: death

Grade 2 complications were reported in 6 out of 9 trials and comprised mainly: urinary tract infections (LSC *n* = 6 [16, 23, 24], VM *n* = 4 [21, 23], RSC *n* = 5 [24], SSF *n* = 5 [21]); postoperative fever (ASC *n* = 4 [19]); wound infection (ASC *n* = 1 [20], RSC *n* = 2 [24]); and pulmonary embolism (ASC *n* = 2 [16, 19]).

The highest grade 3 complication rate was seen after VM (34.2%) [21]. Grade 3 complications were reported in all trials and comprised mainly: bladder lesions in 11 cases (ASC n = 2 [19, 20], SSF *n* = 2 [20, 21], VM *n* = 3 [21], LSC *n* = 5 [16, 17, 23, 24], RSC *n* = 2 [24]); bowel lesions in 3 cases (ASC *n* = 1 [17], LSC *n* = 1 [23], RSC *n* = 1 [24]); severe bleeding in 22 cases (ASC *n* = 3 [17, 19, 20], SSF *n* = 7 [20, 21], LSC *n* = 1 [23], VM *n* = 11 [21, 23]); and mesh problems in 28 cases (ASC *n* = 6 [18–20], LSC *n* = 1 [23], VM *n* = 19 [23], RSC *n* = 2 [24]).

Only 1 study [16] reported a grade 5 complication, which concerned a 79-year-old patient with a fatal bowel perforation after ASC. Supplementary Table 6 (Appendix 2) presents an overview of all complications.

### Intervention details

#### Operating time

The mean operating time could be extracted from 7 studies (*n* = 676) and ranged from 50 to 265 min (Table 3). The shortest operating time was reported for the VM [23], whereas the longest operating time (with and without docking time) was reported for the RSC [24].

#### Blood loss

Mean amount of estimated blood loss during the intervention was extracted from 6 studies (*n* = 598). The mean blood loss ranged from 34 to 306 mL (Table 3). The lowest blood loss was reported for the LSC [17], whereas the ASC was associated with the highest estimated blood loss [20].

#### Duration of hospital stay

The mean duration of hospital stay was reported in 5 studies (*n* = 408) and ranged from 1.4 to 5.4 days (Table 3). The shortest hospital stay was reported for the LSC [17] and the SSF [20]. The ASC was associated with the longest hospital stay [20].

### Recurrence of pelvic organ prolapse

In Table 4 (and Appendix 2 Table 6) follow-up results for all the studies included were presented.

**Table 4:** Technique-specific follow-up results after 1 year

Technique	ASC		LSC	RSC	VM	SSF
	Mesh	Fascia				
Studies	5	1	4	1	3	3
*n*	186	29	154	40	176	165
POP-Q point C						
	[17]: −6.63 (SD 1.35) [16]: −6.7 (SD 1.9) [18]: −9.0 (SD 1.2) Vault to/beyond hymen [20]: 4% (2)	[18]: −8.1 (SD 2.7)	[17]: −6.65 (SD 1.19) [16]: −6.5 (SD 1.6) [20]: −7.48 (SD 2.62) [24]: −10 (range −11 to −5)	[24]: −9 (range −11 to −6)	[20]: −6.11 (SD 2.72) [21]: −5.99 [22]: −6.2 (SD 1.29)	[21]: −4.94 [22]: −3.2 (SD 3.56) Vault to/beyond hymen: [20]: 19% (8)
POP-Q stage < 2						
	[20]: 76% (35/46)^a^ [16]: 66% (19/29) [19]: 91% (41/45)^b^ [18]: 93% (27/29)^b^	[18]: 62% (18/29)^b^ [19]: 61% (30/44)^b^	[16]: 72% (21/29) [20]: 77% (41/53)	–	[20]: 43% (23/55) [21]: 83% [22]: 97%^c^	[20]: 69% (29/42)^a^ [21]: 61% [22]: 35%^c^
Re-operations						
(for POP, incontinence, complications)	[20]: 13% (6/47) [18]: 6% (3/54) [17]: 11% (3/27) [16]: 19% (7/36)	[18]: 4% (2/46)	[20]: 6% (3/53) [17]: 12% (3/26) [16]: 19% (7/37)		[20]: 22% (12/55) [21]: 13% (11/85) [22]: 31% (11/36)	[21]: 5% (4/83) [22]: 18% (6/34) [20]: 27% (13/48)
Re-operations for POP						
	[20]: 2% (1/47) [18]: 2% (1/54) [17]: 0% (0/27) [16]: 3% (1/37)	[18]: 2% (1/46)	[17]: 4% (1/26) [16]: 11% (4/37) [20]: 0% (0/53)	–	[20]: 5% (3/55) [21]: 1% (1/85) [22]: 0% (0/36)	[20]: 6% (3/48) [21]: 5% (4/83) [22]: 9% (3/34)
Mesh exposure						
	[17]: 0% (0/27) [16]: 0% (0/37) [18]: 2% (1/54)	[18]: 2% (1/54)	[17]: 0% (0/26) [16]: 0% (0/37) [20]: 2% (1/53) [24]: 0% (0/38)	[24]: 5% (2/40)	[20]: 13% (7/55) [21]: 21% (16/79) [22]: 8% (3/36)	
Dyspareunia						
	[20]: 20% (9)	–	–	–	[22]: 6% (2/34)	[20]: 19% (9) [22]: 3% (1/36)
De novo incontinence						
	Any incontinence: [17]: 15% (4/27) UUI: [16]: 8% (3/37) SUI: [20]: 9% (2/22) [16]: 11% (4/37)	–	Any incontinence: [17]: 8% (2/26) UUI: [16]: 5% (2/37) SUI: [16]: 14% (5/37)	–	SUI: [22]: 38% (13/34)	SUI: [20]: 33% (8/24) [22]: 8% (3/36)

*UUI* urge urinary incontinence, *SUI* stress urinary incontinence

^a^Baden–Walker grade 2

^b^≤ POP-Q stage 2

^c^Ba, C of Bp above hymen

#### Point C

All studies reported acceptable results for point C of the POP-Q classification at follow-up. The LSC is associated with the best anatomical result of the apical compartment [17], with point C of the POP-Q classification of −10 cm. The poorest anatomical result of the apical compartment is after a SSF, with point C of −3.2 cm [22]. Halaska et al. [21] reported point C according to POP-Q at −4.94 cm after SSF.

#### POP-Q stage < 2

The success rates vary widely. The lowest scores are reported for the SSF [20–22] and ASC with fascia lata [18, 19], with a range of 35–69%. VM reports one of the lowest anatomical success rates of 43% [23], and the highest success rate of 97% [22].

#### Reoperations for POP

The reoperation rate for POP seems to be the lowest after ASC, with a range of 0–3% and the VM [21–23] with a range of 0–5% [20–22]. All procedures report low reoperation rates for POP, except for the outlier of 11% for LSC [16]. However, in another trial [23], the reoperation rate after the LSC is the lowest (0%). The highest general reoperation rates (for POP, incontinence, and complications) are reported for the VM (13–22%) [21–23] and SSF (5–27% [22, 23].

#### Mesh exposure

The reported mesh exposure rate after a sacrocolpopexy is very low, regardless of the introduction technique of fixation material. Mesh exposure after a sacrocolpopexy ranges from 0 to 5% [16–18, 23, 24], in contrast to the VM, which seems to be associated with an exposure rate of 8–21% after 1-year follow-up [21–23].

#### Dyspareunia

Most trials did not report any significant difference between the investigated interventions; however, in only 2 trials were the numbers of participants given. The dyspareunia rate varied between 3 and 20% [20, 22], and ASC and SSF were associated with the highest dyspareunia rates of 20% and 19% respectively.

#### De novo incontinence

Stress urinary incontinence was most frequently seen after VM (38%) [22] and SSF (33%) [23]. However, most trials did not report data on incontinence.

### Meta-analysis

#### Abdominal sacrocolpopexy vs laparoscopic sacrocolpopexy

##### Estimated blood loss, ASC vs LSC

**Fig. 5:**
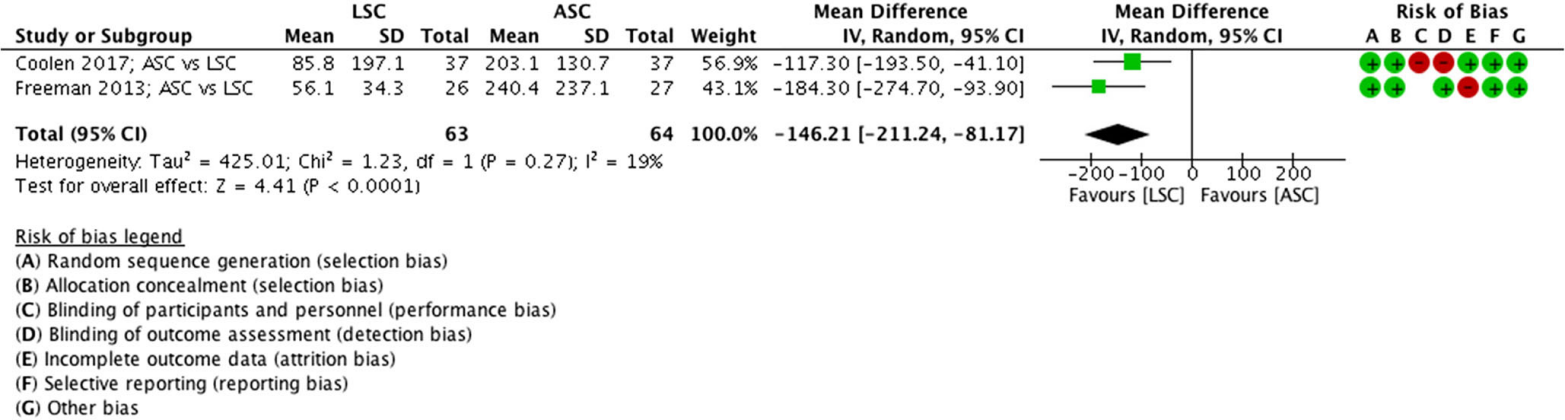
Forest plot of comparison: abdominal sacrocolpopexy (ASC) vs laparoscopic sacrocolpopexy (LSC), outcome 1.1 estimated blood loss

The ASC was associated with less blood loss, compared with LSC (MD −146 ml, 95% CI −211 to −81, 2 RCTs, *n* = 127, I^2^ 19%, high quality evidence; Fig. 5).

##### Operating time, ASC vs LSC

**Fig. 6:**
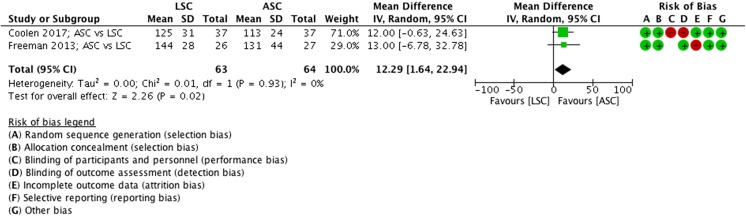
Forest plot of comparison: ASC vs LSC, outcome 1.2 operating time

The operating time of the LSC is shorter than that of ASC (MD 12.3 min, 95% CI −7 to 33, 2 RCTs, *n* = 127, I^2^ 0%, high quality evidence; Fig. 6).

##### Length of hospital stay, ASC vs LSC

**Fig. 7:**
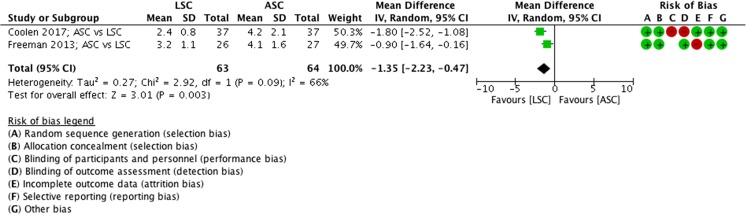
Forest plot of comparison: ASC vs LSC, outcome 1.3 length of hospital stay

Length of hospital stay is shorter after an LSC compared with ASC (MD −1.4 days, 95% CI 1.7 to 23, 2 RCTs, *n* = 127, I2 66%, high-quality evidence; Fig. 7).

##### Complications, ASC vs LSC

**Fig. 8:**
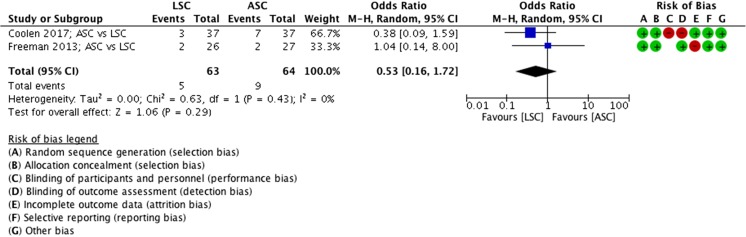
Forest plot of comparison: ASC vs LSC, outcome 1.4 complications

There were more complications after an ASC than after a LSC; however, this result is not significantly different (MD 0.53 events, 95% CI 0.2 to 1.7, 2 RCTs, *n* = 127, I^2^ 0%, high-quality evidence; Fig. 8). There were 5 reported complications in the LSC group versus 9 in the ASC group.

##### Reoperations (for POP), ASC vs LSC

**Fig. 9:**
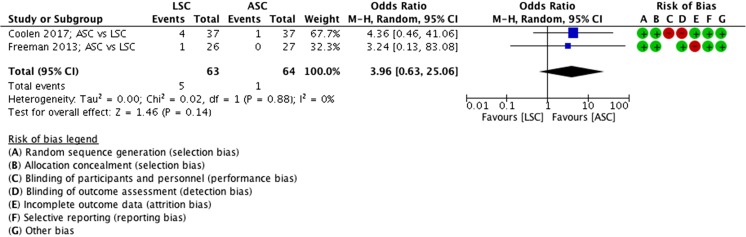
Forest plot of comparison: ASC vs LSC, outcome 1.5 reoperations (for POP)

There was no statistically significant difference between reoperations for POP between ASC and LSC; however, fewer reoperations were seen in the ASC group (MD 4.0 events, 95% CI 0.6 to 25, 2 RCTs, *n* = 127, I^2^ 0%, high-quality evidence; Fig. 9). In the LSC group, 5 reoperations were performed versus 1 in the ASC group.

##### POP-Q point C (at 1 year), ASC vs LSC

**Fig. 10:**
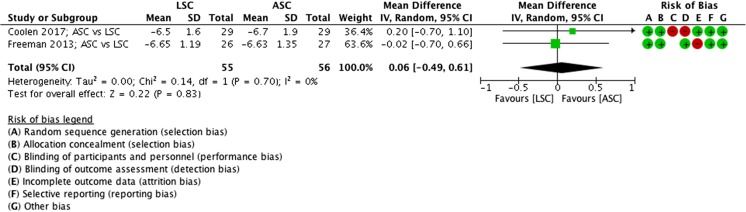
Forest plot of comparison: ASC vs LSC, outcome 1.6 point C (at 1 year)

No differences were seen in POP-Q point C 1 year after an ASC of LSC (MD 0.06 cm, 95% CI −0.49 to 0.61, 2 RCTs, *n* = 127, I^2^ 0%, high-quality evidence; Fig. 10).

##### PGI-I (at 1 year), ASC vs LSC

**Fig. 11:**
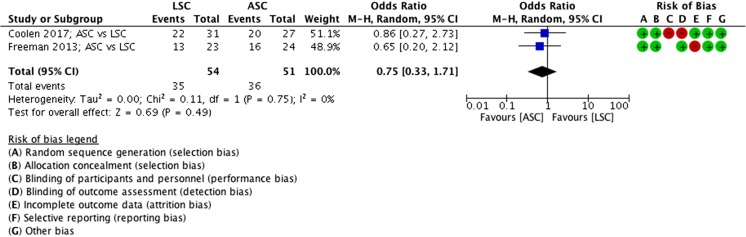
Forest plot of comparison: ASC vs LSC, outcome 1.7 PGI-I (at 1 year)

No differences were seen in participants who scored “much better” and “very much better” on the PGI-I questionnaire, 1 year after an ASC of LSC (MD 0.75 participants, 95% CI 0.33 to 1.71, 2 RCTs, *n* = 127, I^2^ 0%, high-quality evidence; Fig. 11).

#### Sacrospinal fixation vs transvaginal mesh

##### Complications, SSF vs VM

**Fig. 12:**
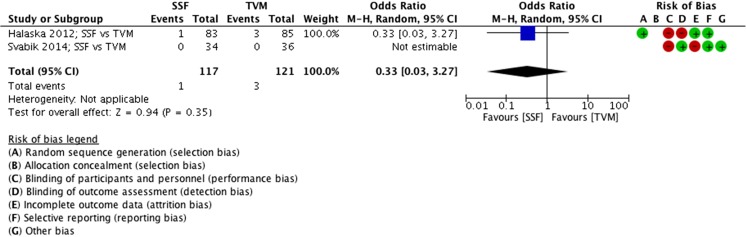
Forest plot of comparison: SSF vs VM, outcome 2.1 complications

The complication rate of SSF compared with VM was not significantly different, as the SSF group reported 1 complication and the VM group 3 (MD 0.33 events, 95% CI 0.03 to 3.27, 2 RCTs, *n* = 238, I^2^ n/a, medium-quality evidence; Fig. 12).

##### Reoperations (for POP), SSF vs VM

**Fig. 13:**
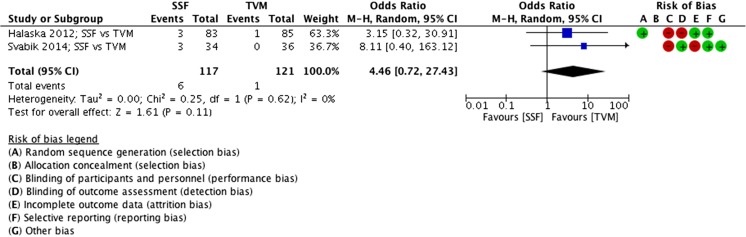
Forest plot of comparison: SSF vs VM, outcome 2.2 reoperations (for POP)

Fewer reoperations for POP were seen in the VM group, compared with SSF; however, this was not significantly different (MD 4.5 events, 95% CI 0.72 to 27.43, 2 RCTs, *n* = 238, I^2^ 0%, medium-quality evidence; Fig. 13). Six reoperations were described in the SSF group versus 1 in the VM group.

## Discussion

### Main findings

We performed a systematic review and meta-analysis, combined with a network plot, to compare the objective and subjective outcome of VVP treatments and to determine the most effective treatment. Ranges for objective success rates for the therapies of VVP were wide and the heterogeneity of the outcome measures of the included trials was large. Therefore, a network meta-analysis was not possible.

All surgical techniques resulted in good subjective results, and with no statistically significant differences between the techniques compared, with the exception of the comparison between VM and LSC. LSC is associated with a higher satisfaction rate. Sacrocolpopexy (laparoscopic, robotic, and abdominal) resulted in the best anatomical results, followed by VM. However, the ranges of the anatomical outcome of VM were large. The poorest results are described for SSF, which also correlates with the higher reoperation rate for POP. Most overall reoperations (for complications, recurrent prolapse, and incontinence) were seen after VM. Most complications (grades 2–5) were reported after ASC, VM, and RSC.

Although differences are negligible, the LSC seems to be the technique with the best results. However, all techniques have proved to be effective; therefore, a standard treatment for VVP could not be given according to this review.

### Strengths and limitations

We performed a systematic review and meta-analyses of all randomized controlled trials available on the topic of the treatment of VVP. Although 9 RCTs were included in this review, a treatment of outspoken preference for VVP could not be determined, owing to the large heterogeneity of the trials. Therefore, a network meta-analysis could not be performed because of the lack of a common reference intervention (standard treatment) and the many different comparisons of all the VVP treatments. However, a network plot was constructed to illustrate the geometry of the network and we pooled several data to perform a meta-analysis (Fig. 4).

Many treatment comparisons have been made, using all the different measurement tools and outcomes. To compare treatment options, standard treatment needs to be uniform, using the same measurement tools and outcomes. These measurement tools should be in line with the recommendations of the ICS/IUGA [26]. Initiators of future trials should be aware of this heterogeneity and need to choose carefully the treatments to compare and outcomes. Not only a reference intervention, but also the recommendation of validated and accepted questionnaires would be helpful. The POP-Q classification should be used for the anatomical outcome. Complications can be recorded in a systematic way by using the CTS classification system as advised by the IUGA/ICS [26] or the Clavien–Dindo complication classification [13]. Success should be defined according to the composite score of Barber et al. (recurrent pelvic organ prolapse beyond the hymen in the apical compartment, with bothersome bulge symptoms, and reinterventions) [25]. Unfortunately, not all included trials used the POP-Q classification to evaluate the anatomical outcome, and the subjective outcome was measured by many different questionnaires, some not even validated. Also, success was defined in many different ways. The same arguments count as for the standardization of complications. All trials report complications in their own way, which could result in a risk of bias, because authors can choose to include or exclude complications at will. Registration of complications should be recorded in a systematic way and described before the start of a trial. However, we structured the trial results and performed meta-analyses to compare the treatments included if possible.

### Interpretation of important outcome measurements

#### Anatomical objective outcome

All trials reporting on success had different definitions of success (Table 1). As different success rate definitions were used, these data are difficult to interpret. Therefore, we extracted data from all publications to look for identical outcome measures (Table 4 and Appendix 2 Table 6). Data for point C from the POP-Q was available for almost all trials. Another anatomical outcome measurement was success defined as POP-Q stage 2 or lower. It has to be taken into account that the definitions based on prolapse stage were also different (“no prolapse POP-Q stage >2,” “no prolapse POP-Q stage ≥ 2,” “Baden–Walker classification,” “position of the vault in relation to the hymen,” and “POP-Q stage of any compartment, or some specific compartment”). Nevertheless, the POP-Q stage data have to be interpreted with caution again. Furthermore, not all papers report on other compartments, whereas some techniques are prone to resulting in recurrences in other compartments, even as the possibility of anatomical overcorrection of compartments after some procedures. In addition, the anatomical result does not completely reflect the patient satisfaction correctly, as described by Barber et al. [25]. Therefore, other parameters such as subjective outcome are also very important.

#### Subjective outcome

All trials used different definitions and measurement tools (Table 2). This makes interpretation difficult. As mentioned before, it would be preferable if the ICS/IUGA could recommend validated questionnaires that can be used in future trials, to compare subjective results. Unfortunately, we were unable to extract any identical data about subjective outcome, to make the comparison easier. Also, we were not able to extract enough data from the composite outcome recommended by Barber et al. [25].

#### Reoperation

Because of the heterogeneity of the subjective and objective outcome measurements used in the trials, reoperations for POP can be a good reflection of the patient’s satisfaction and anatomical result. All trials report low reoperation rates for POP, with an outlier of 11% for LSC in 1 trial [16]. Based on these data we can conclude cautiously that all techniques are effective, with the best results for ASC (0–3%) and VM (0–5%). However, the highest general reoperation rates (for POP, incontinence, and complications), and a wide range of anatomical outcomes, are reported for VM [21–23]. Different techniques are available for fixating the transvaginal mesh for apical suspension. Others show better results of VM, depending on which technique was used (for example, the AMS Elevate system; AMS, Minnetonka, MN, USA) [27, 28].

#### Complications and mesh exposure

The highest complication rate was described for VM (31.6% Clavien–Dindo grade 3). These complications were often associated with mesh exposure, which correlate with the highest exposure rates for the VM technique (8–21%). Mesh exposure reported in the literature ranges from 3.2 to 17% depending on the treated compartment [27, 29–31]. Although the exposure rate of 21% seems to be exceptionally high compared with other studies, women treated with VM should be informed about the chance of exposure and the use of VM should be considered very well.

Serious adverse events and mesh-related complications should also be taken into account for the LSC. The FDA recently issued a public health notification on the use of mesh in surgery for vaginal prolapse treatment. This, however, concerns the use of vaginal meshes for the treatment of vaginal prolapse, as opposed to abdominal mesh. According to our review, the mesh exposure rate ranged from 0 to 5%, with a maximum follow-up time of 5 years. However, higher exposure rates of 10.5% are reported after ASC [32]. Nevertheless, the follow-up time of this trial was 7 years, which could be an explanation for the lower rate in this review. Although mesh complications need to be taken into account in the decision regarding which treatment is to be performed, the prevalence of these complications is lower in LSC than in VM.

The ASC was the only technique associated with a Clavien–Dindo grade 5 complication. This is very rare, but unacceptable for elective surgery. Therefore, surgeons and patients should be aware of the complexity of this abdominal procedure.

## Conclusion

A comparison of techniques was difficult because of heterogeneity; therefore, a network meta-analysis was not possible. All techniques have proved to be effective and the reported differences between the techniques were negligible. Therefore, a standard treatment for VVP could not be given according to this review.

## Electronic supplementary material

Appendix 1 (DOCX 16 kb)

Appendix 2 (DOCX 80 kb)

Appendix 3 (DOCX 93 kb)
