# Supplementary material for: The treatment of post-hysterectomy vaginal vault prolapse: a systematic review and meta-analysis
Source: Int Urogynecol J. 2017 Oct 16;28(12):1767–83. doi: 10.1007/s00192-017-3493-2 (PMC5705749; doi:10.1007/s00192-017-3493-2)
Supplement: Supplementary file 1 — (DOCX 16 kb) [file 192_2017_3493_MOESM1_ESM.docx]

**Appendix 1. Full electronic search strategy PubMed (MEDLINE) and Embase.**

Search strategy PubMed (MEDLINE)

1. vault prolapse*[tiab]
2. (Therapy/Narrow[filter]) AND (#1)
3. systematic[sb] AND (#1)
4. (#2 OR #3)

Search strategy Embase

1. exp vaginal vault prolapse
2. limit 1 to (evidence based medicine or consensus development or meta-analysis or outcomes research or "systematic review")
3. limit 2 to ("therapy (maximizes sensitivity)" or "therapy (maximizes specificity)" or "therapy (best balance of sensitivity and specificity)")
4. 2 or 3
