# Supplementary material for: The treatment of post-hysterectomy vaginal vault prolapse: a systematic review and meta-analysis
Source: Int Urogynecol J. 2017 Oct 16;28(12):1767–83. doi: 10.1007/s00192-017-3493-2 (PMC5705749; doi:10.1007/s00192-017-3493-2)
Supplement: Supplementary file 2 — (DOCX 80 kb) [file 192_2017_3493_MOESM2_ESM.docx]

**Appendix 2: additional tables of outcomes**

**Table 5: complications**

| **Complication*** | **Grade 1** | **Grade 2** | **Grade 3** | **Grade 4** | **Grade 5** |
| --- | --- | --- | --- | --- | --- |
| **Maher 2004**  ASC vs SSF | ASC: 0% (0/47)  SSF: 2.1% (1/48)   - 1 vaginal pain of unknown origin | ASC: 2% (1/47)   - 1 wound infection   SSF: 0% (0/48) | ASC: 10.6% (5/47)   - 1 blood transfusion - 1 cystostomy - 2 incisional hernia procedures - 1 mesh rejection   SSF: 6.3% (3/48)   - 1 blood transfusion - 1 cystostomy - 1 rectovaginal hematoma | ASC: 0% (0/47)  SSF: 0% (0/48) | ASC: 0% (0/47)  SSF: 0% (0/48) |
| **Culligan 2005**  ASC:  Fascia lata vs polypropylene mesh | Fascia: 0% (0/46)  Mesh: 0% (0/54) | Fascia: 4.3% (2/46)   - 2 postoperative fever   Mesh: 5.6% (3/54)   - 2 postoperative fever - 1 postoperative pulmonary embolism | Fascia: 10.9% (5/46)   - 5 wound breakdown   Mesh: 22.2% (12/54)   - 2 ileus - 8 wound breakdown - 2 exposure of graft - 1 intraoperative bladder injury - 1 blood transfusion | Fascia: 0% (0/46)  Mesh: 0% (0/54) | Fascia: 0% (0/46)  Mesh: 0% (0/54) |
| **Tate 2011**  ASC:  fascia lata vs polypropylene mesh | Fascia: 0% (0/44)  Mesh: 0% (0/45) | Fascia: 0% (0/44)  Mesh: 0% (0/45) | Fascia: 2.3% (1/44)   - 1 graft exposure   Mesh: 4.4% (2/45)   - 2 graft exposure   Removal of 1 mesh required partial bowel resection  resulting in a colostomy.  The other one was removed laparoscopically and developed necrotizing fasciitis postoperatively at the umbilical port site. | Fascia: 0% (0/44)  Mesh: 0% (0/45) | Fascia: 0% (0/44)  Mesh: 0% (0/45) |
| **Freeman 2013**  ASC vs LSC | ASC: 0% (0/27)  LSC: 0% (0/26) | ASC: 0% (0/27)  LSC: 0% (0/26) | ASC: 7.4% (2/27)   - 1 detachment of an area of mesentery of the small bowel which required a resection of 10 cm of small bowel - 1 excessive bleeding of sacrum which required haemostatic bone wax   LSC: 7.7% (2/26)   - 1 opening of the vagina - 1 bladder injury | ASC: 0% (0/27)  LSC: 0% (0/26) | ASC: 0% (0/27)  LSC: 0% (0/26) |
| **Coolen 2017**  ASC vs LSC | ASC: 0% (0/37)  LSC: 0% (0/36) | ASC: 2.7% (1/37)   - 1 pulmonary embolism   LSC: 5.6% (2/36)   - 1 wound infection - 1 pyelonefritis | ASC: 13.5% (5/37)   - 2 wound dehiscence - 3 ileus   LSC: 5.6% (2/36)   - 1 bladder lesion - 1 bleeding | ASC: 0% (0/37)  LSC: 0% (0/36) | ASC: 2.7% (1/37)   - 1 fatal bowel perforation   LSC: 0% (0/36) |
| **Maher 2011**  LSC vs VM | LSC: 0% (0/53)  VM 0% (0/55) | LSC: 3.8% (2/53)   - 2 urinary tract infection   VM: 10.9% (6/55)   - 3 urinary tract infection - 1 infected pelvic hematoma that settled with intravenous antibiotics - 2 mesh exposure (vaginal estrogen therapy) | LSC: 9.4% (5/53)   - 1 cystostomy - 1 small bowel enterotomy - 1 blood transfusion - 1 mesh exposure (vaginal estrogen therapy + mesh exposure correction) - 1 trocar hernia   VM: 18.2% (10/55)   - 1 blood transfusion - 5 mesh exposure (vaginal estrogen therapy + mesh exposure correction) - 4 mesh contractions | LSC: 0% (0/53)  VM 0% (0/55) | LSC: 0% (0/53)  VM 0% (0/55) |
| **Paraiso 2011**  LSC vs RSC | LSC: 0% (0/33)  RSC: 2.9% (1/35)   - 1 corneal abrasion | LSC: 9.1% (3/33)   - 3 urinary tract infection   RSC: 28.6% (10/35)   - 5 urinary tract infection - 2 wound infection - 3 abdominal wall pain necessitating trigger point injection | LSC: 9.1% (3/33)   - 2 cystostomy - 1 abscess   RSC: 22.9% (8/35)   - 2 cystostomies - 1 enterotomy - 2 small bowel obstruction - 2 mesh exposure (one was from tension-free vaginal tape) - 1 abscess | LSC: 0% (0/33)  RSC: 0% (0/40) | LSC: 0% (0/33)  RSC: 0% (0/40) |
| **Halaska 2012**  SSF vs VM | SSF: 2.7% (2/73)   - 2 dyspareunia - 3 pelvic pain   VM: 0% (0/79) | SSF: 6.9% (5/73)   - 5 LUT infections   VM: 8.9% (7/79)   - 1 LUT infection - 6 mesh exposure (treated with local estrogen therapy | SSF: 9.6% (7/73)   - 1 bladder perforation - 6 severe bleeding   VM: 31.6% (25/79)   - 3 bladder perforation - 10 severe bleeding - 1 hematoma - 1 abscess - 10 mesh exposure treated by surgical resection | SSF: 0% (0/73)  VM: 0% (0/79) | SSF: 0% (0/73)  VM: 0% (0/79) |
| **Svabik 2014**  SSF vs VM | SSF: 0% (0/34)  VM: 2.8% (1/36)   - 1 mesh exposure, asymptomatic + treated conservatively | SSF: 0% (0/34)  VM: 0% (0/36) | SSF: 0% (0/34)  VM: 5.6% (2/36)   - 2 mesh exposure, resected during TVT-O procedure | SSF: 0% (0/34)  VM: 0% (0/36) | SSF: 0% (0/34)  VM: 0% (0/36) |

ASC = abdominal sacrocolpopexy, LSC = laparoscopic sacrocolpopexy, SSF = sacrospinous fixation, RSC = robotic sacrocolpopexy, VM = total vaginal mesh

*Grade 1: requires no treatment; grade 2: requires drug therapy; grade 3: requires a procedure or intervention; grade 4: IC/ICU organ or system dysfunction; grade 5: death

**Table 6: follow-up results after one year**

| **Author** | **Maher**  **2004** | **Culligan**  **2005** | **Tate**  **2011** | **Freeman**  **2013** | **Coolen**  **2017** | **Maher**  **2011** | **Paraiso**  **2011** | **Halaska**  **2012** | **Svabik**  **2014** |
| --- | --- | --- | --- | --- | --- | --- | --- | --- | --- |
| **Comparison** | ASC vs SSF | ASC:  fascia vs polypropylene | ASC:  fascia vs polypropylene | ASC vs LSC | ASC vs LSC | LSC vs VM | LSC vs RSC | SSF vs VM | SSF vs VM |
| **POP-Q point C** |  | | | | | | | | |
|  | Vault to/beyond hymen:  ASC: 2 (4%)  SFF: 8 (19%) | No difference | Fascia: -8.1  (SD 2.7)  Mesh: -9.0  (SD 1.2) | LSC: -6.65  (SD 1.19)  ASC: -6.63  (SD 1.35) | LSC: -6.5  (SD 1.6)  ASC: -6.7  (SD 1.9) | LSC: -7.48  (SD 2.62)  VM: -6.11  (SD 2.72) | LSC: -10  (range -11 to -5)  RSC: -9  (range -11 to -6) | SSF: -4.94  VM: -5.99 | SSF: -3.2  (SD 3.56)  VM: -6.2  (SD 1.29) |
| **POP-Q stage<2** |  |  |  |  |  |  |  |  |  |
|  | *ASC: 35/46 (76%)  SSF: 29/42 (69%) | ‡Fascia: 30/44 (61%)  Mesh: 41/45 (91%) | ‡Fascia: 18/29 (62%)  Mesh: 27/29 (93%) | not mentioned | LSC: 21/29 (72%)  ASC: 19/29 (66%) | LSC: 41/53 (77%)  VM: 23/55 (43%) | not mentioned | SSF: 61%  VM: 83% | ∫SSF: 35%  VM: 97% |
| **Re-operations for POP** |  | | | | | | | | |
|  | ASC: 1/47 (2%)  SSF: 3/48 (6%) | not mentioned | Fascia: 1/46 (2%)  mesh: 1/54 (2%) | LSC :1/26 (4%)  ASC: 0/27 (0%) | LSC: 4/37 (11%)  ASC: 1/37 (3%) | LSC: 0/53 (0%)  VM: 3/55 (5%) | not mentioned | SSF: 4/83 (5%)  VM: 1/85 (1%) | SSF: 3/34 (9%)  VM: 0/36 (0%) |
| **Mesh exposure** |  | | | | | | | | |
|  | not mentioned | not mentioned | Fascia: 1/54 (2%)  Mesh: 1/54 (2%) | LSC: 0/26 (0%)  ASC: 0/27 (0%) | LSC: 0/37 (0%)  ASC: 0/37 (0%) | LSC 1/53 (2%)  VM 7/55 (13%) | LSC: 0/38 (0%)  RSC: 2/40 (5%) | VM: 16/79 (21%) | VM: 3/36 (8%) |
| **Dyspareunia** |  | | | | | | | | |
|  | ASC: 9 (20%)  SSF: 9 (19%) | not mentioned | not mentioned | no difference | no difference according to questionnaire | no difference according to questionnaire | not mentioned | no difference | SSF: 1/36 (3%)  VM: 2/34 (6%) |
| **De novo incontinence** |  | | | | | | | | |
|  | SUI:  ASC: 2/22 (9%)  SSF: 8/24 (33%) | not mentioned | not mentioned | Any incontinence:  LSC: 2/26 (8%)  ASC: 4/27 (15%) | UUI:  LSC: 2/37 (5%)  ASC: 3/37 (8%)  SUI:  LSC 5/37 (14%)  ASC 4/37 (11%) | not mentioned | not mentioned | no difference | SUI:  SSF: 3/36 (8%)  VM: 13/34 (38%) |

ASC = abdominal sacrocolpopexy, LSC = laparoscopic sacrocolpopexy, SSF = sacrospinous fixation, RSC = robotic sacrocolpopexy, VM = total vaginal mesh

UUI = urge urinary incontinence, SUI = stress urinary incontinence

* Baden Walker grade 2, ‡= ≤ POP-Q stage 2, ∫= Ba, C of Bp to above hymen
