# Supplementary material for: The treatment of post-hysterectomy vaginal vault prolapse: a systematic review and meta-analysis
Source: Int Urogynecol J. 2017 Oct 16;28(12):1767–83. doi: 10.1007/s00192-017-3493-2 (PMC5705749; doi:10.1007/s00192-017-3493-2)
Supplement: Supplementary file 3 — (DOCX 93 kb) [file 192_2017_3493_MOESM3_ESM.docx]

**Appendix 3. Characteristics of included studies; systematic assessment**

**Maher 2004**

| ***Methods*** | Multi-centre (2 sites, Australia), RCT. 2-year follow-up |
| --- | --- |
| ***Participants*** | Inclusion criteria: women with symptomatic posthysterectomy vaginal vault prolapse that extended to or beyond the introitus.  Exclusion criteria: women who had undergone a previous sacral colpopexy or had a significantly foreshortened vagina. |
| ***Interventions*** | Abdominal sacral colpopexy with Prolene mesh (N=47) versus vaginal sacrospinous colpopexy (N=48) |
| ***Outcomes*** | Primary and secondary outcomes were not specified.  Outcomes:  - subjective success (no symptomps of prolapse);  - objective success (no vaginal prolapse ≥ grade 2 Baden Walker classification at any vaginal site during a Valsalva maneuver);  - patient satisfaction on a visual analog scale (VAS) from 0 to 100  - SUI  - overactive bladder  - voiding dysfunction  - constipation  - obstructed defecation  - fecal incontinence  - sexual activity  - dyspareunia  - quality of life: SF-36, UDI, IIQ  - re-interventions due to complications  - complications  - costs (US dollars) |

| ***Risk of bias*** | | |
| --- | --- | --- |
| **Bias** | **Authors' judgement** | **Support for judgement** |
| Random sequence generation (selection bias) | Low risk | Once agreeing to participate in the study, women were randomly allocated to the vaginal or the abdominal approach. Women with SUI were stratified to ensure equal representation in each group. Randomization lists were computer generated and held by the nonsurgical coauthor. |
| Allocation concealment (selection bias) | High risk | Not described. |
| Blinding of participants and personnel (performance bias)  *All outcomes* | High risk | Not described. |
| Blinding of outcome assessment (detection bias)  *All outcomes* | High risk | Not described. |
| Incomplete outcome data (attrition bias)  *All outcomes* | High risk | 7% of the women did not complete meaningful follow-up and approximately 10% did not complete full review. Reasons for loss to follow-up not described. |
| Selective reporting (reporting bias) | Low risk | No selective outcome reporting. |
| Other bias | Not applicable | None. |

**Culligan 2005**

| ***Methods*** | Single-centre (USA), RCT. 1-year follow-up |
| --- | --- |
| ***Participants*** | Inclusion criteria: women with posthysterectomy vaginal vault prolapse scheduled for sacral colpopexy through the Division of Urogynecology and Reconstructive Pelvic Surgery at the University of Louisville Health Sciences Center.  Exclusion criteria: not described. |
| ***Interventions*** | Abdominal sacral colpopexy with cadaveric fascia lata (N=50) or polypropylene mesh (N=50) |
| ***Outcomes*** | Primary outcome: anatomic failure (prolapse ≥ POP-Q stage II)  Secondary outcomes:  - subjective outcome (quality of life, PISQ, ISI, constipation severity score, defecation diary, pain); not described in results  - complications |

| ***Risk of bias*** | | |
| --- | --- | --- |
| **Bias** | **Authors' judgement** | **Support for judgement** |
| Random sequence generation (selection bias) | Low risk | A computerized blocked randomization scheme (using blocks of 8) was constructed to determine the type of material that would be used for the sacral colpopexy. |
| Allocation concealment (selection bias) | Low risk | The researchers received only a stack of 104 opaque, numbered, sealed envelopes, each containing the assignment for the subject number on the outside of the envelope. Each patient's envelope was opened immediately before her surgery. |
| Blinding of participants and personnel (performance bias)  *All outcomes* | Low risk | The following procedures were used to maintain the double-blinding within the study: the actual material used for a given colpopexy appeared in only 2 places: the dictated operative note and the master list of the randomization scheme. The master list for the randomization scheme generated was held by the statistician. All patients were made aware of the importance of their not knowing which material had been used for their surgery. During each surgery, members of the surgical team were reminded not to reveal the nature of the material used to the patient. |
| Blinding of outcome assessment (detection bias)  *All outcomes* | Low risk | To avoid the possibility of patient steering, the researchers were unaware of the block size. The certified clinical research nurse who collected all of the data throughout the study period did not have access to the dictated operative notes or the master list of the randomization scheme. |
| Incomplete outcome data (attrition bias)  *All outcomes* | Unclear | Although the randomization scheme called for an even 50/50 breakdown, there were 46 patients who received fascia and 54 who received polypropylene mesh. The reason for the discrepancy was that 4 patients randomized to receive fascia were actually given mesh because of a transient shortage of the Tutoplast material. A total of 89 patients (45 in the mesh group and 44 in the fascia group) returned for their 1-year follow-up visits. No reasons for loss to follow-up mentioned. |
| Selective reporting (reporting bias) | Low risk | No selective outcome reporting. |
| Other bias | High risk | Data presentation unclear, selective reporting of subjective outcome, complication minimally reported, re-interventions not reported. |

**Maher 2011**

| ***Methods*** | Single-centre (Australia), RCT. 2-year follow-up |
| --- | --- |
| ***Participants*** | Inclusion criteria: consecutive women referred to Wesley, Royal Brisbane’s and Mater tertiary referral Urogynaecology unit with symptomatic stage 2 or greater (point C ≥ -1 POP-Q) vaginal vault prolapse.  Exclusion criteria:  - age < 18 years  - inability to comprehend questionnaires, to give informed consent or to return for review  - vault prolapse < stage 2  - unable to undergo general anesthesia  - BMI >35  - 5 previous laparotomies  - prior sacral colpopexy or vaginal mesh prolapse procedure  - vaginal length < 6 cm |
| ***Interventions*** | Laparoscopic sacrocolpopexy (N=53) versus total vaginal mesh with Total Prolift (Gynecare, Ethicon) (N=55) |
| ***Outcomes*** | Primary outcome: objective success rates at POP-Q sites Aa, Ba, C, Bp and Ap defined as less than -1 cm individually and as a total.  Secondary outcomes:  - patient satisfaction (VAS)  - quality of life (Australian Pelvic Floor Questionnaire (APFQ), Kings College Pelvic Organ Prolapse quality of life (P-QoL)  - complications  - reoperations |

| ***Risk of bias*** | | |
| --- | --- | --- |
| **Bias** | **Authors' judgement** | **Support for judgement** |
| Random sequence generation (selection bias) | Low risk | After completion of study consent, research support staff were telephoned and allocation to the laparoscopic or vaginal surgery group from randomization list that were computer generated by the study statistician, stratified for urodynamic stress incontinence (SUI and occult SUI) with full allocation concealment, was completed. |
| Allocation concealment (selection bias) | Unclear | Method of allocation concealment not described. |
| Blinding of participants and personnel (performance bias)  *All outcomes* | Unclear | Full allocation concealment. Other blinding not described. |
| Blinding of outcome assessment (detection bias)  *All outcomes* | High risk | Not described. |
| Incomplete outcome data (attrition bias)  *All outcomes* | Unclear | Described in the randomized trial flow diagram. No reasons for loss to follow-up described. |
| Selective reporting (reporting bias) | Low risk | No selective outcome reporting. |
| Other bias | Not applicable | None. |

**Paraiso 2011**

| ***Methods*** | Single-centre (USA), RCT. 1-year follow-up |
| --- | --- |
| ***Participants*** | Inclusion criteria:  - posthysterectomy vaginal apex prolapse at Pelvic Organ Prolapse Quantitative stages 2–4  - age ≥ 21 years  - desired laparoscopic surgical management  Exclusion criteria:  - contra-indication for general anesthesia  - history of prior sacrocolpopexy  - suspicious adnexal masses  - history of pelvic inflammatory disease  - morbid obesity (BMI ≥ 40)  - history of prior or concomitant surgery for rectal prolapse |
| ***Interventions*** | Laparoscopic sacrocolpopexy (N=38) versus robot-assisted sacrocolpopexy (N=40) |
| ***Outcomes*** | Primary outcome: time from initial incision to skin closure (total operative time)  Secondary outcomes:  - total time the patient was in the operating room (operative room time)  - time that the patient was under anesthesia from beginning of induction to extubation (anesthesia time)  - time for specific parts of the case including robotic cart docking (docking time only applicable to robot)  - complications  - pain scale (visual analog scale, amount of narcotic and NSAIDs used during hospitalization  - activity scale (Activity Assessment Scale, return to normal activities (visual analog scale))  - anatomic outcome (POP-Q)  - quality of life (PFDI-20, PFIQ-7, PISQ, EQ-5D)  - costs (US dollars) |

| ***Risk of bias*** | | |
| --- | --- | --- |
| **Bias** | **Authors' judgement** | **Support for judgement** |
| Random sequence generation (selection bias) | Low risk | Particpants were assigned randomly in a 1:1 ratio to one of two treatment groups. Treatment allocation was determined by a computer-generated randomization schedule with random block sizes (two to six) and stratified by surgeon. |
| Allocation concealment (selection bias) | Low risk | Treatment assignments were placed in consecutively numbered, opaque-sealed envelopes that were opened by the surgery scheduler immediately before scheduling the case because each procedure required different equipment that needed to be known before the day of surgery (on average this occurred 42 days before surgery). |
| Blinding of participants and personnel (performance bias)  *All outcomes* | Low risk | Patients were blinded to their treatment assignment. Operating room and healthcare providers responsible for intraoperative and postsurgical care were informed not to discuss treatment assignment during the preoperative discussion or the postoperative period. |
| Blinding of outcome assessment (detection bias)  *All outcomes* | Low risk | Research staff administering and collecting the study questionnaires and outcomes were blinded to the participant’s treatment group for the entire duration of the study. |
| Incomplete outcome data (attrition bias)  *All outcomes* | Unclear | Follow-up displayed in flow chart. Not a lot of loss to follow-up, but reasons for loss to follow-up are not described. |
| Selective reporting (reporting bias) | Low risk | No selective outcome reporting. |
| Other bias | Not applicable | None. |

**Tate 2011**

| ***Methods*** | Single-centre (USA), RCT. 5-year follow-up of Culligan 2005. |
| --- | --- |
| ***Participants*** | Inclusion criteria: women with post hysterectomy vaginal vault prolapse scheduled for abdominal sacrocolpopexy through the Division of Urogynecology and Reconstructive Pelvic Surgery at the University of Louisville Health Sciences Center.  Exclusion criteria: not described. |
| ***Interventions*** | Initial trial of Culligan 2005: abdominal sacral colpopexy with cadaveric fascia lata (N=50) or polypropylene mesh (N=50). 5-year follow-up: cadaveric fascia lata (N=29) and polypropylene mesh (N=29). |
| ***Outcomes*** | Primary outcome: anatomic failure (prolapse ≥ POP-Q stage II)  Secondary outcomes:  - clinical relevant success, defined as a combination of a subjective component (no bulge or prolapse symptoms), an anatomic component (prolapse < POP-Q stage II) and the need for surgical re-treatment of pelvic organ prolapse  - subjective outcome (interview by research nurse; quality of life, PISQ, ISI, constipation severity score, defecation diary, pain)  - re-treatment (interview by research nurse)  - complications |

| ***Risk of bias*** | | |
| --- | --- | --- |
| **Bias** | **Authors' judgement** | **Support for judgement** |
| Random sequence generation (selection bias) | Low risk | As previously reported, a computerized blocked randomization scheme (using blocks of 8) was constructed to determine the type of material that would be used for the sacrocolpopexy. The master list for the randomization scheme was held by the statistician. |
| Allocation concealment (selection bias) | Low risk | The researchers received opaque, numbered, sealed envelopes, each containing the assignment for the subject number on the outside of the envelope. To avoid the possibility of patient steering, the researchers were unaware of the block size. Each patient’s envelope was opened immediately before surgery. |
| Blinding of participants and personnel (performance bias)  *All outcomes* | Low risk | The design of the study was a "double-blinded randomized study". Only the surgical team was aware of the subject’s assignment to a graft type. Throughout the initial 1-year as well as the 5-year follow-up period, all preoperative and postoperative outcome measures for both studies were obtained by a single, masked, clinical research nurse. The subjects were told by the surgeon which material they received after they completed the 1-year follow-up period. At the time of the 5-year follow-up visit, the subjects were asked not to reveal the type of graft material that had been used. Therefore, the clinical research nurse remained masked despite the fact that subjects were aware of their material type at the 5-year point. The principal investigator for the 5-year study did not perform any of the original surgeries and was masked to the type of graft material used. |
| Blinding of outcome assessment (detection bias)  *All outcomes* | Low risk | Described above. |
| Incomplete outcome data (attrition bias)  *All outcomes* | High risk | Fifty-eight of the 100 subjects (58%) returned for their 5-year visit—29/54 from the polypropylene mesh group and 29/46 from the fascia lata group. Eleven of the 100 subjects (11%) returned only questionnaires and therefore were not included in any of the analyses due to their lack of POP-Q examinations at 5 years. Thirty-one subjects did not respond, declined participation, and were lost to follow-up or had died of causes unrelated to the surgery. The follow-up rate did not significantly differ between treatment groups. The subjects who did not return for the 5-year follow-up were slightly older (60±12 years) than those who returned for follow-up (58±9 years), but the difference was not significant (p=0.44). |
| Selective reporting (reporting bias) | High risk | Primary and secondary outcome measurements are described in the methods and the same measurements are shown in the results. |
| Other bias | High risk | Data presentation unclear, selective reporting of subjective outcome. |

**Halaska 2012**

| ***Methods*** | Multi-centre (Czech Republic), RCT. 1-year follow-up |
| --- | --- |
| ***Participants*** | Inclusion criteria: all patients scheduled for surgery for vaginal prolapse with objectively verified symptoms by POP-Q ≥ stage II.  Exclusion criteria:  - pelvic malignancy  - age < 18 years  - history of radiotherapy of the pelvis  - requiring hysterectomy |
| ***Interventions*** | Sacrospinous fixation (N=83) versus transvaginal mesh (Prolift; Gynecare/Ethicon) (N=85) |
| ***Outcomes*** | Primary outcome: prolapse recurrence (prolapse ≥ POP-Q stage II)  Secondary outcomes:  - quality of life (PISQ, UIQ, CRAIQ, POPIQ)  - complications  - reoperations (for prolapse recurrences) |

| ***Risk of bias*** | | |
| --- | --- | --- |
| **Bias** | **Authors' judgement** | **Support for judgement** |
| Random sequence generation (selection bias) | Low risk | Random allocation to surgery was performed by one person (O.S.) using a computer-generated random sequence of zeros and ones. |
| Allocation concealment (selection bias) | Unclear | Not described. |
| Blinding of participants and personnel (performance bias)  *All outcomes* | High risk | Not described. |
| Blinding of outcome assessment (detection bias)  *All outcomes* | High risk | Not described. |
| Incomplete outcome data (attrition bias)  *All outcomes* | Low risk | Loss to follow-up and data analysis are clearly described in the study's flow chart. |
| Selective reporting (reporting bias) | Low risk | No selective outcome reporting. |
| Other bias | Unclear | Data presentation unclear. |

**Freeman 2013**

| ***Methods*** | Multi-centre (UK), RCT. 1-year follow-up |
| --- | --- |
| ***Participants*** | Inclusion criteria: patients with symptomatic and bothersome vault prolapse ≥ POP-Q grade II with or without concomitant cystocele and rectocele.  Exclusion criteria:  - medically unfit for sacrocolpopexy  - requirement for concomitant pelvic or stress urinary incontinence surgery (those patients who had “occult” stress urinary incontinence, i.e. on prolapse reduction only, were not excluded)  - BMI>35  - previous abdominal or vaginal vault prolapse surgery |
| ***Interventions*** | Abdominal sacrocolpopexy (N=27) versus laparoscopic sacrocolpopexy (N=26) |
| ***Outcomes*** | Primary outcome:  - anatomic outcome: POP-Q point C  - subjective outcome: Patient Global Impression of Improvement (PGI-I)  Secondary outcomes:  - operating time  - the distance at which the mesh was attached down the anterior and posterior walls  - blood loss, haemoglobin  - the need for concomitant posterior repair  - pain assessment (VAS, opiate use)  - length of stay  - convalescence  - return to usual activities  - quality of life (P-QOL, SF-36)  - complications  - new onset/occult urinary incontinence (Birmingham bowel and lower urinary tract symptoms questionnaire)  - further surgery by 1 year |

| ***Risk of bias*** | | |
| --- | --- | --- |
| **Bias** | **Authors' judgement** | **Support for judgement** |
| Random sequence generation (selection bias) | Low risk | Patients were randomised to a particular surgeon, but the randomisation was blocked to ensure similar numbers of patients for each surgeon/procedure. Randomisation to either of the two surgical procedures was by computer allocation, co-ordinated by the study statistician. The allocation to this design was generated using Genstat (8th edition). Randomisation took place after the patient had been placed on the appropriate consultant’s waiting list and informed consent had been given (for both procedures). |
| Allocation concealment (selection bias) | Low risk | Described above. |
| Blinding of participants and personnel (performance bias)  *All outcomes* | Unclear | It was not possible to fully “blind” the patient to the type of operation because of the incisions. However, the ward staff who supervised analgesic requirements were blinded. The operation note was placed in a sealed envelope in the patient’s notes and was only to be opened in an emergency. The abdomen was “bandaged”, as for ASCP, following both procedures until the day of discharge. This was aimed at preserving blinding when the post-operative assessments were carried out and was only to be broken if there were complications requiring removal of the bandage. |
| Blinding of outcome assessment (detection bias)  *All outcomes* | Low risk | At the 12-week visit the POP-Q examination was performed by the research fellow or surgeon before any other assessments to minimize bias and maintain blinding. To achieve this, the patient was positioned in the left lateral position by the research nurse and the examining doctor then carried out the POP-Q assessment blind to the identity of the patient. |
| Incomplete outcome data (attrition bias)  *All outcomes* | High risk | There is a patient flow, but the exclusions and loss to follow-up are not described. |
| Selective reporting (reporting bias) | Low risk | Primary and secondary outcome measurements are described in the methods and the same measurements are shown in the results. |
| Other bias | Not applicable | None. |

**Svabik 2014**

| ***Methods*** | Single-centre (Czech Republic), RCT. 1-year follow-up |
| --- | --- |
| ***Participants*** | Inclusion criteria:  - posthysterectomy patients with at least two-compartment prolapse (with affected apical/vault compartment, ≥ POP-Q stage II  - suffering from symptoms of prolapse  - requesting pelvic floor reconstructive surgery  - diagnosed with a complete unilateral or bilateral avulsion injury  Exclusion criteria:  - patients with prolapse and uterus in place  - patients without levator ani avulsion  - patients not requesting pelvic floor surgery |
| ***Interventions*** | Sacrospinous vaginal colpopexy (N=34) versus transvaginal mesh (Prolift Total; Gynecare/Ethicon) (N=36) |
| ***Outcomes*** | Primary outcome: anatomic failure based on clinical and ultrasound assessment, defined as:  - Ba, C or Bp at the hymen or below ánd  - on translabial ultrasound as bladder descent to 10 mm or more below the lower margin of the symphysis pubis on maximum Valsalva  Secondary outcomes:  - continence status assessment (clinical stress test and subjective evaluation (ICIQ-SF, PISQ-12, POPDI, UDI, CRADI))  - complications  - reoperations (SUI, prolapse recurrence) |

| ***Risk of bias*** | | |
| --- | --- | --- |
| **Bias** | **Authors' judgement** | **Support for judgement** |
| Random sequence generation (selection bias) | Unclear | The randomization process was carried out by computer, using patient hospitalization numbers, the night before surgery. Patients were informed about their allocation after the procedure. |
| Allocation concealment (selection bias) | Unclear | Described above. |
| Blinding of participants and personnel (performance bias)  *All outcomes* | High risk | Patients were informed about their allocation after the procedure. |
| Blinding of outcome assessment (detection bias)  *All outcomes* | Low risk | These postoperative clinical examinations were performed by a single examiner, who had not been involved with the surgical procedures and was unaware of a patient’s procedure at the start of the assessment, although in some cases group allocation became obvious due to palpable mesh, mesh erosion or visibility of an SSF suture. |
| Incomplete outcome data (attrition bias)  *All outcomes* | High risk | There is a patient flow, but no details about the follow-up. |
| Selective reporting (reporting bias) | Low risk | Primary and secondary outcome measurements are described in the methods and the same measurements are shown in the results. |
| Other bias | Not applicable | None. |

**Coolen 2017**

| ***Methods*** | Multi-centre (The Netherlands), RCT. 1-year follow-up |
| --- | --- |
| ***Participants*** | Inclusion criteria: women with a history of hysterectomy presenting with symptomatic vaginal vault prolapse, with or without concomitant cystocele and rectocele, who choose to undergo surgery.  Exclusion criteria:  - previous surgical correction of a vault prolapse  - contra-indication for a surgical intervention because of general physical condition |
| ***Interventions*** | Abdominal sacrocolpopexy (N=37) versus laparoscopic sacrocolpopexy (N=37) |
| ***Outcomes*** | Primary outcome: functional outcome (UDI)  Secondary outcomes:  - other subjective outcomes (DDI, IIQ, PGI-I, questions about sexuality)  - peri-operative outcomes (procedure time, blood loss, hospital stay, complications, re-interventions (incontinence, prolapse))  - anatomical outcome (POP-Q)  - composite outcome of success, defined as no prolapse beyond the hymen, no bothersome bulge symptoms, no repeat surgery or pessary use for recurrent prolapse within 12 months |

| ***Risk of bias*** | | |
| --- | --- | --- |
| **Bias** | **Authors' judgement** | **Support for judgement** |
| Random sequence generation (selection bias) | Low risk | After written informed consent was given, randomisation was performed by an independent research secretariat located in Amsterdam after a phone call or e-mail by the coordinating investigator. The treatment allocation was done by opaque sealed envelopes in a 1:1 ratio to either LSC or ASC. Women received a case number at randomisation to treat their data anonymously. |
| Allocation concealment (selection bias) | Low risk | Described above. |
| Blinding of participants and personnel (performance bias)  *All outcomes* | High risk | Participants and physicians were not blinded for the intervention, as blinding is impossible between the very different types of incision. |
| Blinding of outcome assessment (detection bias)  *All outcomes* | High risk | Observers and physicians were not blinded for the intervention.The observer was an independent researcher, who had not performed the surgery. |
| Incomplete outcome data (attrition bias)  *All outcomes* | Low risk | At 12 months follow up there were 14 questionnaires missing, of which 11 participants (15.5%) were unwilling to complete the questionnaires, two did not receive the intervention and one patient died five days after the intervention due to a complication of the intervention. The number of missing questionnaires is equal in both groups. All non-responders were contacted by telephone and most of them explained that they were doing excellent which was a reason not to return the questionnaires. Patient characteristics of responders and non-responders were comparable. |
| Selective reporting (reporting bias) | Low risk | Primary and secondary outcome measurements are described in the methods and the same measurements are shown in the results. |
| Other bias | Not applicable | None. |
